# Exercise-Associated Risk After Spontaneous Coronary Artery Dissection: Implications From Highly Active Patients

**DOI:** 10.1016/j.jacadv.2026.102714

**Published:** 2026-04-02

**Authors:** Ioannis Skalidis, Julius Jelisejevas, Giacomo Maria Cioffi, Stephane Cook, Mario Togni

**Affiliations:** HFR – Fribourg Cantonal Hospital and University, Fribourg, Switzerland

Spontaneous coronary artery dissection (SCAD) remains a leading cause of acute coronary syndromes in younger, otherwise healthy individuals, in whom physical exertion is frequently implicated as a potential trigger yet incompletely understood. In this context, Battenberg et al. report a prospective registry analysis of highly active individuals with SCAD, identifying a distinct presentation phenotype without excess long-term recurrence or adverse events.^1^ By leveraging a large, well-curated SCAD registry, the authors convincingly demonstrate that HAIs represent a distinct clinical phenotype, characterized by fewer traditional cardiovascular risk factors, more frequent exercise-associated presentation, and different angiographic patterns, without excess long-term recurrence or major adverse cardiac events.

The study raises several points that merit further discussion and may help refine both mechanistic understanding and clinical guidance. First, the observation that HAIs more commonly present with non–ST-segment elevation myocardial infarction and less frequently with ST-segment elevation myocardial infarction, left main, or multivessel SCAD invites speculation regarding pathophysiological modifiers. Could exercise-related vascular or autonomic adaptations (such as enhanced collateral circulation, altered shear stress responses, or attenuated sympathetic surges) contribute to less extensive dissections or earlier symptom recognition in this group? Additional imaging or physiological correlates might help disentangle biological protection from behavioral factors such as heightened interoceptive awareness.

Second, exercise-associated cardiac arrest, although rare, occurred disproportionately among highly active patients, with most events clustering around high-intensity or unfamiliar exertion. How should clinicians reconcile this low absolute risk with the clear association between vigorous exertion and arrhythmic events? Would stratification by type, intensity, or novelty of physical activity provide a more nuanced framework than current binary activity classifications when counseling SCAD survivors?

Third, HAIs were less likely to remain on beta-blockers and statins during follow-up despite similar outcomes. This finding raises questions about long-term medical therapy in this subgroup. Are medication discontinuations primarily patient-driven due to perceived side effects and performance limitations or clinician-driven based on perceived lower risk? Prospective evaluation of symptom burden, exercise tolerance, and adherence may clarify whether tailored pharmacologic strategies are warranted.

Finally, although recurrence rates did not differ by activity status, physical activity was assessed only at the time of SCAD. How might post-SCAD changes in exercise behavior influence long-term outcomes, psychological recovery, and cardiovascular health? Longitudinal, time-dependent analyses of activity patterns could be particularly informative in shaping evidence-based return-to-sport recommendations.

Overall, this important study advances the field by reframing SCAD in highly active individuals not as a uniformly high-risk group, but as a phenotype requiring individualized risk assessment and counseling. We anticipate that continued work in this area will further refine safe, patient-centered exercise guidance after SCAD.
